# Design Verification Testing for Prefilled Syringes: A Structured Best-Practice Framework

**DOI:** 10.3390/pharmaceutics18050559

**Published:** 2026-04-30

**Authors:** Bettine Boltres, Olga Laskina, Brett Andrejko

**Affiliations:** West Pharmaceutical Services, Stolberger Str. 21–41, 52249 Eschweiler, Germany; olga.laskina@westpharma.com (O.L.); brett.andrejko@westpharma.com (B.A.)

**Keywords:** prefilled syringes, design verification testing, combination product development, EDDOs, sample strategy, risk-based development

## Abstract

**Background:** Prefilled syringes (PFSs) are increasingly used for self- and assisted administration of high-value parenterals, yet design verification (DV) planning remains challenging due to overlapping drug, device, and combination product expectations, as well as limited harmonization of device components. To the best of our knowledge, there is no publication providing an end-to-end DV approach for developers. This work aims to provide a best-practice template for structuring and justifying DV programs for PFSs, with the explicit intent of improving transparency and offering practical clarity to development teams navigating regulatory and technical complexity. **Methods:** A risk-based DV approach is presented for an exemplary 1 mL long staked needle glass PFS intended for subcutaneous administration of a surrogate solution representative of a high-concentration biologic. The approach starts with the design inputs which were derived from intended use, user requirements, and the drug’s quality target product profile (QTPP), then translated into design outputs including Essential Drug Delivery Outputs (EDDOs). These outputs were proven by executing drug-independent and simulated drug-dependent DV tests using ISO- and pharmacopeia-aligned methods, including defined sampling, and real-time/accelerated aging. **Results:** A best-practice DV approach is presented, including test results across the evaluated functional, mechanical, and integrity endpoints. **Conclusions:** The presented approach provides a transferable DV template linking intended use to acceptance criteria, sample size rationale, and test selection. As a best-practice contribution, it supports more consistent, defensible DV planning for PFSs and may reduce ambiguity in the interface between drug and device development expectations.

## 1. Introduction

Prefilled syringes (PFSs) have become a key driver in the growth of primary container closure and delivery systems for parenteral drug products, accelerated by trends towards self-administration, new biologic entities, home care, and the need to reduce healthcare-associated infections and medication errors [1]. As more patients inject high-value, often lifesaving therapies outside controlled clinical settings, the robustness of PFS design and performance has a direct impact on public health by helping to prevent device-related failures, patient non-adherence, drug recalls, and avoidable treatment interruptions.

There are still sterile injectable recalls linked to container closure issues such as loss of container closure integrity (CCI) and/or contamination, underlining the need for robust design, manufacturing and control strategies for PFSs [2,3,4]. In parallel, there is active debate about the clinical relevance of factors such as silicone oil-associated particles and protein aggregates, certain aspects of analytical approaches (e.g., for extractables and leachables and biocompatibility), or about the relative benefits and risks of traditional glass versus alternative polymer syringes, leading to diverging views on specification settings, material selection and how stringent design verification (DV) needs to be, increasing the complexity of the DV approach.

Within the medical device and combination product development framework, design verification is the process that demonstrates that design outputs meet the predefined design input requirements. It is a core element of design and development controls under 21 CFR 820 and ISO 13485 [5,6], and must be applied to combination products with a device constituent part, including prefilled syringes. Current best practice emphasizes that DV test plans should not be “tick lists” of generic methods but be derived from a holistic, risk-based assessment that considers both drug product and device aspects of the combination product [7].

Despite extensive efforts for standardization of individual test methods for prefilled syringes, the literature provides limited practical guidance on how to structure an end-to-end DV program that is traceable to intended use while remaining defensible at the interface of drug, device, and combination product expectations. This article therefore describes a structured DV approach that derives design inputs and DV tests from intended use and risk-based rationale, and reports the resulting evidence for critical functional, mechanical, and integrity attributes. The novelty of this work is therefore a transferable DV best-practice template that integrates:

1. a traceability logic from intended use and user requirements to Quality Target Product Profile (QTPP)-driven [8] design inputs/outputs;
2. a focused Essential Drug Delivery Output (EDDO) [9] framework to distinguish drug-delivery-critical outputs from other design outputs;
3. a pragmatic, risk-commensurate sample size rationale for mixed variable and attribute endpoints in prefilled syringe DV.

To demonstrate how this template is applied in practice, a real-world PFS case study containing a surrogate solution is used. By presenting this end-to-end logic together with the corresponding results, we aim to provide a transparent, best-practice case study that can support other teams in designing, executing, and interpreting PFS DV programs and reduce ambiguity in acceptance criteria settings and sampling decisions where component-level limits are not harmonized across standards.

### 1.1. Intended Use, User Needs and Use Requirements

Our starting point concerns the intended use, user needs and use requirements which form the basis for both drug and device development. These are expanded into the QTPP, design inputs/outputs and Essential Drug Delivery Outputs (EDDOs) that directly inform the DV test matrix.

The following exemplary case was chosen: a 1 mL long prefilled glass syringe configuration containing fancydemumab at 100 mg/mL, developed for subcutaneous injection by a healthcare professional (HCP; the user). An exemplary intended use statement as it would finally appear in the Instructions For Use (IFU) could read:

“*The prefilled syringe, containing fancydemumab, is intended for the subcutaneous administration to treat rheumatoid arthritis in adult patients, including elderly patients, and is administered by healthcare professionals. It is designed to ensure accurate dosing and reliable, ergonomic handling for trained users, supporting consistent treatment according to the prescribed regimen*.”

Defining the intended use and the exact patient population is critical, as it drives key design choices (e.g., if there is no need for an extended finger flange to support reduced grip strength, finger flange does not need to incorporated) and influences toxicological considerations, such as the analytical evaluation threshold (AET) in biocompatibility and extractables and leachables (E and L) assessments [10].

User needs and user requirements translate the intended use into concrete expectations for product performance from the perspective of healthcare professionals and patients. They must derive from human factor, clinical, dose-ranging studies, patient counseling data (e.g., interviews/training observations) or literature references. User needs describe, in simple language, what must be achievable, whereas user requirements express these needs as specific, measurable and verifiable criteria (Table 1). Literature is available covering quantified and mapped forces that humans can comfortably exert based on sex, percentile, digit, health condition, etc. [11,12].

These user needs/requirements are directly linked to the DV tests and acceptance criteria described later, such as break loose and glide force testing, deliverable/residual volume measurements, and mechanical robustness tests (needle shield pull-off, needle pull-out, flange breakage).

### 1.2. Quality Target Product Profile (QTPP) and Design Inputs

The QTPP within the drug development process “forms the basis of design” of the drug product [8] and includes the intended use (see intended use statement above) and the critical quality attributes (CQAs) that must be achieved to ensure the desired quality, safety, and efficacy. It is important to note that the QTPP is iteratively refined throughout development, with the first truly finalizable version typically emerging around Phase II once clinically informed parameters such as dose strength, dose volume, and administration requirements have been sufficiently evaluated, recognizing that these criteria may continue to evolve as additional data are generated.

A vial presentation typically involves limited product/container interactions beyond basic compatibility and CCI. In contrast, a prefilled syringe introduces multiple drug/device part interface interactions that can directly affect product quality, safety, efficacy, and functionality, so a clear, robust QTPP is essential to translate clinical needs into the correct syringe design. The QTPP must also derive from human factor or clinical studies or literature references. Each defined parameter should be supported by a clear, documented rationale to ensure it is scientifically and technically defensible. Some exemplary QTPP elements relevant for syringe development are listed in Table 2.

The prefillable syringe (as the device constituent part) must be selected to ensure that the drug can meet these QTPP targets.

### 1.3. Design Inputs, Design Outputs and Essential Drug Delivery Outputs (EDDOs)

Building on this, the design inputs for the device constituent part are defined. They encompass:

- the intended use statement and defined user population;
- user needs and user requirements;
- QTPP attributes and associated CQAs;
- critical material attributes (CMAs);
- critical process parameters (CPPs) relevant to device performance.

Because these elements are interdependent, they have to be developed in an integrated manner. For example, the viscosity of fancydemumab solution might require further dilution and therefore influence the dose volume, which in turn determines the choice of barrel dimensions and needle gauge. A potential elderly rheumatoid arthritis population informs ergonomics (e.g., large round flange or extended finger flange in case of self-injection) and acceptable force levels (lower than typical in case of self-injection), and CQAs such as aggregate levels constrain material and lubricant choices for barrel, plunger and needle shield. Design inputs must be defined as specific, measurable and traceable into design outputs and the corresponding DV test protocol. Examples of design inputs and outputs relevant for this case study are given in Table 3.

Within this framework, EDDOs represent the small subset of system-level outputs that are most critical for the safe and effective delivery of the drug. They link user needs, risk analysis and QTPP to concrete, measurable performance criteria that must be demonstrated during DV (see Table 4). EDDOs per FDA are “essential for the proper functioning of the device” to deliver the drug (i.e., the intended use of the drug delivery device) [9].

Note that glide force describes the plunger movement within an empty syringe to ensure consistent gliding, while extrusion force describes the force needed to extrude a liquid in a filled syringe. This difference becomes apparent with high viscosity drugs.

A challenge is that regulatory and pharmacopeial sources generally define acceptance criteria primarily at the finished product level, whereas explicit limits for individual syringe components (e.g., glass barrel, plunger stopper, rigid needle shield) are often not prescribed. Consequently, component-level specifications must be jointly established by the supplier and the drug product manufacturer based on risk, intended use and system performance requirements, which increases complexity and may introduce variability and uncertainty in how “fit-for-purpose” is demonstrated across organizations.

Finally, all developed acceptance criteria are assembled in a specification for the device constituent part (example shown in Table 5).

There is no consistent distinction between tests considered as “core” design verification (DV) of device performance and tests that are more material, process, or system qualification-driven (e.g., bioburden, endotoxins, particles, biocompatibility, extractables/leachables, transportation simulation, sterilization validation). In this manuscript, DV is defined narrowly as verification of the functional and mechanical performance tests, whereas the remaining activities would be covered by the respective process validation strategies or material specifications.

All design inputs and outputs and EDDOs must be captured in the risk management plan and evaluated using a formal FMEA, according to ICH Q9 and ISO 14971 [17]. Concerning the FMEA, it should be recognized that the device constituent part suppliers typically manufacture syringes for multiple applications and therefore generally cannot perform a use case- and patient-specific risk assessment without the drug product developer’s intended use context. As part of the design FMEA (dFMEA), potential ways in which the PFS system could fail to meet defined design inputs (e.g., functional, performance, and compliance-related requirements) are systematically identified, the potential impact of each failure is evaluated, plausible causes/mechanisms are assessed, and detection controls are defined. These elements are then used to estimate overall risk and to prioritize design controls, DV test selection, and bracketing conditions for the verification program.

### 1.4. Test Sample Size

The allocated risk priority class derived from the probability of occurrence and the severity of a specific harm then drives the decision of statistical sample sizes, such that the sampling plan is commensurate with the associated risk. Defining DV sample sizes is particularly challenging because risk scoring schemes and the resulting confidence/reliability targets vary across organizations and tests. Also, across the industry it is not always consistently defined whether a given test should be categorized as an attribute or a variable test, and companies apply differing classification approaches. While ISO 11608-1:2022 Annex F can serve as a pragmatic approximation, there is no broadly applicable regulatory guideline prescribing precise DV sample sizes for each test [18]. The most common practical distinction between attribute and variable evaluation is seen with container closure integrity (CCI): deterministic methods (e.g., helium leak) generate continuous leak-rate data, yet acceptance is typically applied as a pass/fail decision against a predefined threshold. Consequently, CCI is often treated as a variable during method development and limit justification, but as an attribute outcome for DV and specification compliance. ISO 11608-1:2022 Annex F provides a table (Table F.1) for sample sizes for attribute testing. For variable endpoints, sample size is additionally determined considering the tolerance factor k (which depends on one- vs. two-sided limits and increases when variability is estimated from the sample), distributional assumptions (normal vs. non-normal), and the need to represent key sources of variation (lots, timepoints) with statistically independent units.

In practice, a commonly applied convention (based on company internal standard procedures, depending on risk appetite and differing between companies) is a 95% confidence/95% reliability (“95/95”) target, which corresponds to sample sizes of a minimum of 20 for variable testing and 59 for attribute demonstrations.

### 1.5. Testing Strategy

Design verification testing is generally performed in two phases: drug-independent testing, which may be carried out at any stage and is typically performed upon device part selection, and drug-dependent testing, which requires the actual drug product (or a surrogate solution if justifiable) to evaluate potential interactions with the device part. For drug-dependent testing, the final commercial prefilled syringe should be used to capture any effects from the fill/finish process, such as plunger setting [19].

Table 6 provides an overview of the classification for the current approach.

Samples tested to support design verification should be representative of the final product design and manufactured under Good Manufacturing Practice (GMP) commercial conditions. This includes having undergone shipping simulation before testing. Upon justification, certain drug-dependent tests can be performed using a surrogate solution.

It is a regulatory expectation to support shelf life with real-time stability data under the intended storage conditions, and to complement these data with accelerated studies in line with ICH stability principles (e.g., ICH Q1A(R2) [21,22]). In practice, real-time studies should extend through the proposed expiry period (commonly 24–36 months), while accelerated studies provide supportive, time-compressed evidence and help elucidate degradation pathways. For drug products intended for refrigerated storage (2–8 °C), ICH Q1A(R2) specifies long-term storage at 5 ± 3 °C and an accelerated condition of 25 ± 2 °C/60% RH ± 5% RH. For clarity, it should be noted that “stability” and “shelf life” refer not only to the chemical and physical stability of the drug product, but also to the functional performance stability of the delivery device, such that aged devices are tested to confirm continued performance throughout the labeled shelf life.

### 1.6. Platforming, Design Space and Quality Management System

The industry is clearly trending towards implementing platforming approaches wherever possible to streamline processes. The proposed DV template is readily extendable to a formulation platform strategy by establishing a standardized procedure. Within a defined formulation platform, the DV template can be combined with a structured bracketing strategy to efficiently cover the majority of relevant parameter combinations within the qualified design space. Specifically, worst-case brackets (e.g., upper viscosity at cold temperature, extremes of concentration/ionic strength, and representative surfactant levels) can be selected based on risk assessment and their expected impact on EDDO-linked performance such as injection forces, injection time, and delivered volume. This approach can streamline design verification by reducing redundant testing across nominal conditions while still providing objective evidence that performance requirements are met across the platform envelope. If a new molecule is formulated within this qualified framework, the DV template and supporting evidence can be leveraged with a structured formulation gap and risk assessment, supplemented by targeted confirmatory testing under worst-case and shelf life conditions. Conversely, formulation excursions outside the platform framework necessitate the re-justification and re-verification of affected EDDO-critical endpoints, particularly extrusion force and injection time.

Similarly, variations in design space and design inputs govern the extent to which the established test matrix, acceptance criteria, and sampling rationale can be directly leveraged. If a new program remains within the qualified framework of critical inputs (e.g., viscosity/temperature range, needle configuration, siliconization, plunger setting process windows, materials and interface tolerances, and storage/distribution conditions), the template can be applied largely unchanged with a bridging justification. Conversely, excursions toward the boundaries or outside the qualified design space may alter failure modes and margins for EDDO-linked performance (e.g., deliverable volume and injection forces), thereby necessitating the re-justification of limits and bracketing. In such cases, a structured gap and risk assessment should be used to identify which endpoints require confirmatory or full DV testing to re-establish objective evidence under the new configuration, formulation, and shelf life conditions.

While companies implement different quality management systems and documentation structures, the proposed DV template is QMS-agnostic because it is based on traceability and risk-based justification rather than company-specific workflows. Differences in QMS primarily affect ownership, approval pathways, risk classification thresholds, and change-control triggers, which in turn influence bracketing decisions, sample size rationale, and the level of supporting evidence required.

## 2. Materials and Methods

The syringe system configuration in scope of this case study is:

- Syringe: West, Synchrony™ S1 PFS system, 1 mL long staked needle, 29 G TW ½”, sprayed-on silicone oil medical grade 1000 cSt, EtO sterilized, West Waterford, Ireland.
- Needle shield: West, design rigid 4144, formulation 7028/55, EtO sterilized, West Waterford, Ireland.
- Plunger: West, 1 mL long NovaPure^®^, formulation 4023/50 with FluroTec^TM^ barrier film and B2-Coating, steam sterilized, West Waterford, Ireland.
- Surrogate solution (fancydemumab): 100 mg/mL lysozyme (Sigma Aldrich, Atlanta, GA, USA), L-histidine/L-histidinehydrochloride buffer (20 mM; Sigma Aldrich, Saint Louis, MO, USA), 5% trehalose (Thermo Fisher Scientific, Pittsburg, PA, USA), 150 mM NaCl (Thermo Fisher Scientific, Pittsburg, PA, USA), 0.02% PS80 (Sigma Aldrich, Atlanta, GA, USA), pH 6.
- Fill volume: 1.05 mL.

Drug-independent testing was performed based on Table 6, and data were collected up to 1 year, both accelerated and real-time aging. Due to the limited amount of the fancydemumab surrogate solution, the simulated drug-dependent tests had to be performed using a sample size that was around a factor of four less than the actual sample size used for drug-independent testing. Drug-dependent data were collected for up to 6 months, both accelerated and real-time aging. Some data were not yet available at the time of writing. USP <697> specifies five samples to be used.

Table 7 gives an overview of the test matrix. Abbreviations for timepoints measured are: T0, 3 months real time (T3MRT), 3 months accelerated aging (T3MAA), 6 months real time (T6MRT), 6 months accelerated aging (T6MAA), 1-year real time (T1YRT), 1-year accelerated aging (1YAA).

The analytical method validations ([14], Section 6.1 in [18]) are out of the scope of this article, as most methods are described in the corresponding ISO standards. Precision, including repeatability and reproducibility studies, were performed to statistically verify that the analytical method’s measurement system is precise, consistent across operators, and suitable for generating reliable validation and release data [22,23]. Filling and plunger placement were performed using both a Bausch and Ströbel SVP 4600 (Bausch and Ströbel, Ilshofen, Germany) and a Colanar FSV 1253 machine (Colanar, Deep River, CT, USA). The plunger in filled syringes was placed using the vacuum-assisted placement method.

Force testing was performed using different models of Instron with axial modules and equipped with various load cells from 10 N to 2000 N to accommodate a range of forces being tested.

Samples aging at real time were stored at ambient room temperature. Surrogate solution samples were stored under monitored but not controlled humidity while drug-independent samples were stored under controlled humidity. Samples undergoing accelerated aging were stored at 40 ± 2 °C and 75% ± 5 RH conditions.

Needle shield pull-off force was measured according to ISO 11040-4:2024 standards, Annex G.6, method 2. The syringe was placed in the fixturing of a force testing machine. The needle shield and syringe body were both secured in the fixturing and were pulled apart. The maximum force corresponding to the needle shield pull-off force was recorded.

Needle pull-out force was measured according to ISO 11040-4:2024 standards, Annex G.1. The syringe was placed in the fixturing of a force testing machine. The needle and syringe body were both secured in the fixturing and were pulled apart. The maximum force corresponding to the needle pull-out force was recorded.

Needle penetration was measured according to ISO 11040-4:2024 standards, Annex F. The syringe was placed in the fixturing of a force testing machine. A test foil was affixed in a fixture under the needle. The needle penetrated the foil as force was recorded. The penetration force profile was recorded and analyzed; the maximum value was compared with the needle penetration specification.

Break loose and glide forces were measured according to ISO 11040-4:2024 standards, Annex E. The plunger was placed into an empty syringe and then this assembly was placed in the fixturing of a force testing machine. A plunger rod was inserted and depressed while the forces were recorded as the plunger traveled down the barrel. The initial maximum force required to move the plunger was recorded as the break loose force and the maximum force required to sustain the plunger movement was recorded as the maximum glide force.

Break loose and extrusion forces were measured similarly to ISO 11040-4:2024 standards, Annex E. The plunger was placed into the surrogate filled syringe and then this assembly was placed in the fixturing of a force testing machine. A plunger rod was inserted and depressed while the forces were recorded as the plunger traveled down the barrel. Initial maximum force required to move the plunger was recorded as the break loose force and the maximum force required to sustain the plunger movement was recorded as the maximum extrusion force.

The tightness test (blue dye) was performed similarly to ISO 11040-4:2024 standards, Annex H. Syringes were filled with water at a nominal volume and the plunger was placed. Syringe systems were then placed in a chamber filled with a solution of methylene blue dye. The pressure in the chamber was then reduced by 270 mbar and held for 10 min. After that, the pressure was returned to atmospheric pressure and the syringes were left immersed for another 30 min. The syringes were then removed, rinsed from external dye, and dried. After that, they were inspected for any traces of dye solution ingress.

Closure system liquid leakage was measured according to ISO 11040-4:2024 standards, Annex G.2. The syringes were filled with water and surrogate solution, respectively, between 1/3 and 2/3 of the nominal fill volume, and then a pressure of 110 kPa was applied for 5 s. After that, the sample was examined for signs of leakage.

Container closure integrity was evaluated as recommended in ISO 11040-8:2016, USP <382> and USP <1207> [24]. Helium leak methodology was used. The syringes were filled with water and surrogate solution, respectively, placed into the fixturing, and 100% helium gas flow was applied. The system was monitored under vacuum to determine the leak rate of helium. The plunger–syringe body interface was tested separately from the front closure interface.

Liquid leakage at the plunger was evaluated according to ISO 7886-1:2015, Annex D. The syringes were filled with water and surrogate solution, respectively, to the nominal capacity, the plunger was placed, and the closure end of the syringes was sealed. An axial pressure of 300 kPa was applied to the plunger stopper by the plunger rod, along with an addition of a sideways force of 0.25 N. The pressure was maintained for 30–35 s and the syringe system was then examined for leakage past the plunger seal.

Flange breakage resistance was measured according to ISO 11040-4:2024 standards, Annex C.1. The syringe was placed in the fixturing of a force testing machine. The loading pin was used to apply a force to the syringe while the force required to break the syringe flange was recorded.

To measure deliverable volume the syringe was first filled with 1.05 mL surrogate solution, and the plunger was placed. Deliverable volume was measured based on USP <697>. The liquid was expelled into a dry weighed beaker by slowly and constantly depressing the plunger. The resulting liquid was weighed and then the result was converted to volume using the density of the liquid.

Residual volume was measured according to ISO 7886-1:2017 standards, Annex C. The syringe assembly (syringe barrel and plunger) was weighed dry. Then it was filled, and the plunger was placed. After that, the liquid was expelled by slowly and constantly depressing the plunger. The resulting empty syringe assembly was weighed again. The weight gain of the syringe assembly after filling and emptying, relative to the initial dry weight, was then converted to volume using the density of the liquid.

## 3. Results

### 3.1. Needle Shield Pull-Off Force

**Figure 1:**
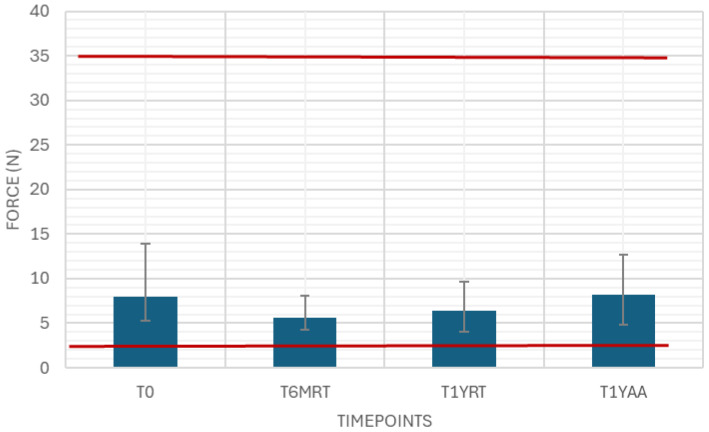
Needle shield pull-off force. Variable drug-independent test, 40 samples, specification: ≥2 N and ≤35 N.

The needle shield pull-off force was measured as a variable drug-independent test with 40 samples at each timepoint. The results were in between the limits of 2 and 35 N (Figure 1).

### 3.2. Needle Pull-Out Force

**Figure 2:**
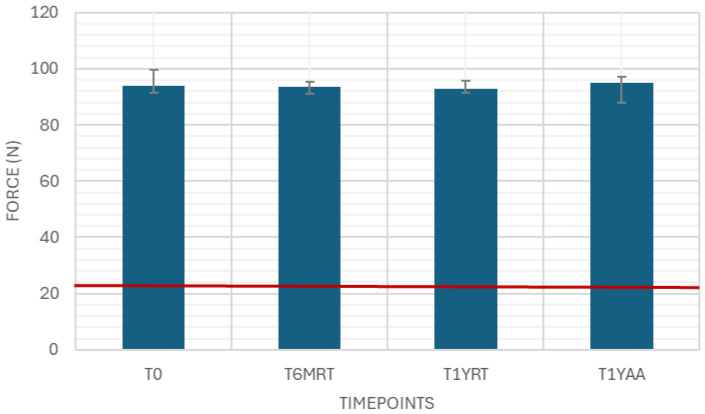
Needle pull-out force. Variable drug-independent test, 40 samples, specification: ≥22 N.

The needle pull-out force was measured as a variable drug-independent test with 40 samples. The results were well above the limit of 22 N (Figure 2).

### 3.3. Needle Penetration Force

**Figure 3:**
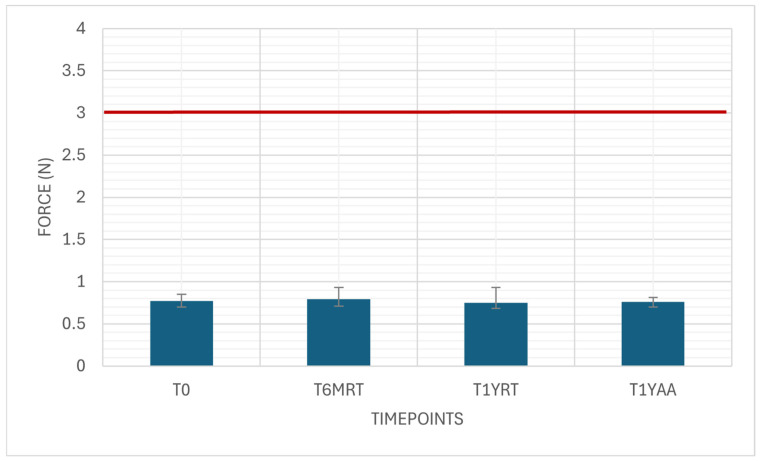
Needle penetration force. Variable drug-independent test, 35 samples, specification: ≤3 N.

The needle penetration force was measured as a variable drug-independent test with 35 samples. The results were well below the limit of 3 N (Figure 3).

### 3.4. Flange Breakage Resistance

**Figure 4:**
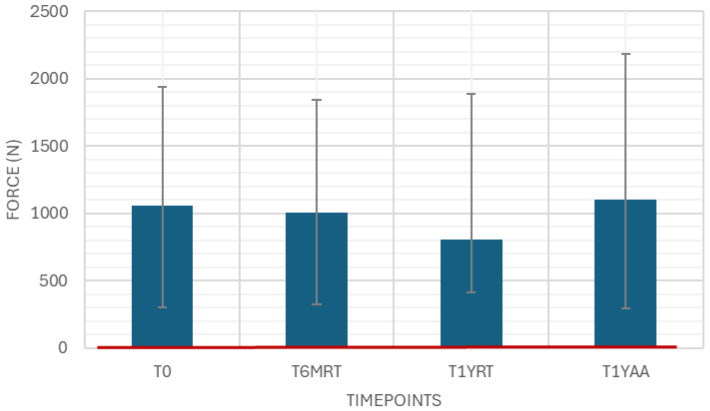
Flange breakage resistance. Variable drug-independent test, 40 samples, specification: ≥35 N.

The flange breakage resistance was measured as a variable drug-independent test with 40 samples. The results were well above the limit of 35 N (Figure 4).

### 3.5. Break Loose and Glide/Extrusion Force

**Figure 5:**
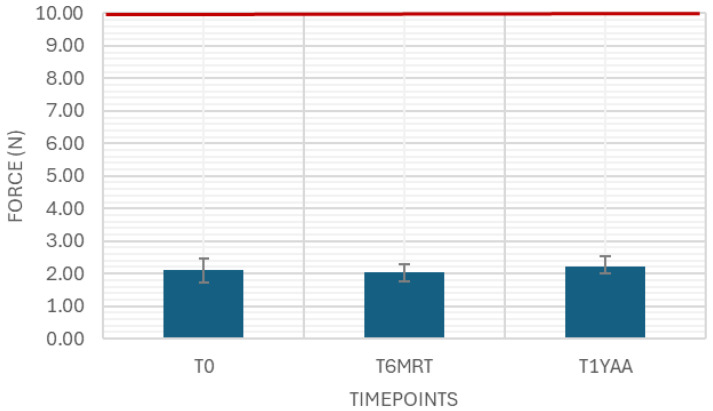
Break loose force. Variable drug-independent test, 40 samples, specification: ≤10 N.

**Figure 6:**
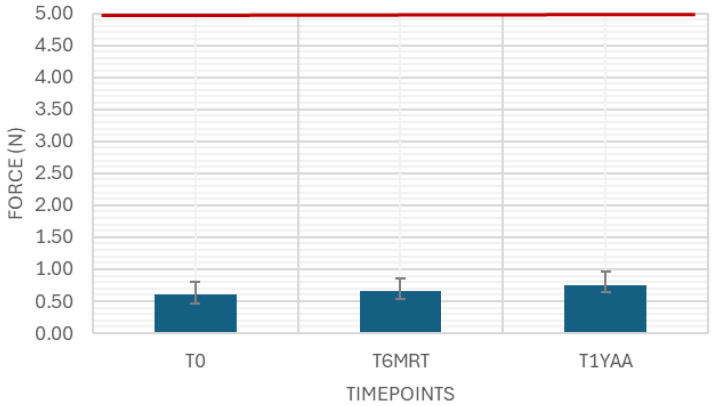
Glide force. Variable drug-independent test, 40 samples, specification: ≤5 N.

The break loose and glide force were measured as variable drug-independent tests with 40 samples. The results were well below the limit of 10 N and 5 N, respectively (Figure 5 and Figure 6).

**Figure 7:**
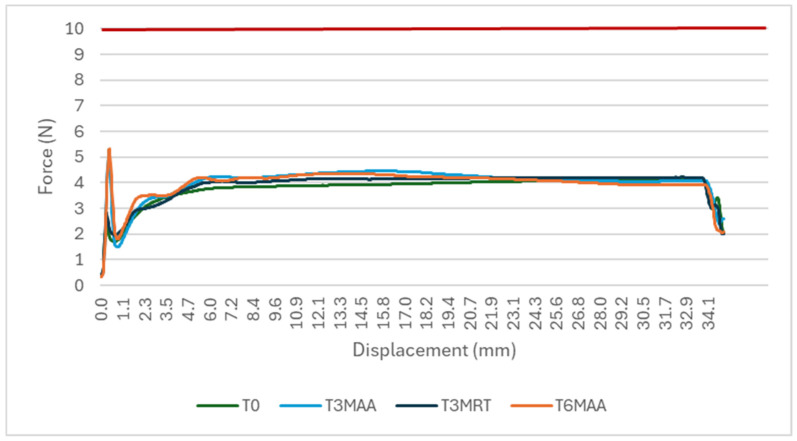
Break loose and extrusion. Variable simulated drug-dependent test, 10 samples at each timepoint, specification: break loose ≤10 N, extrusion force ≤20 N.

**Figure 8:**
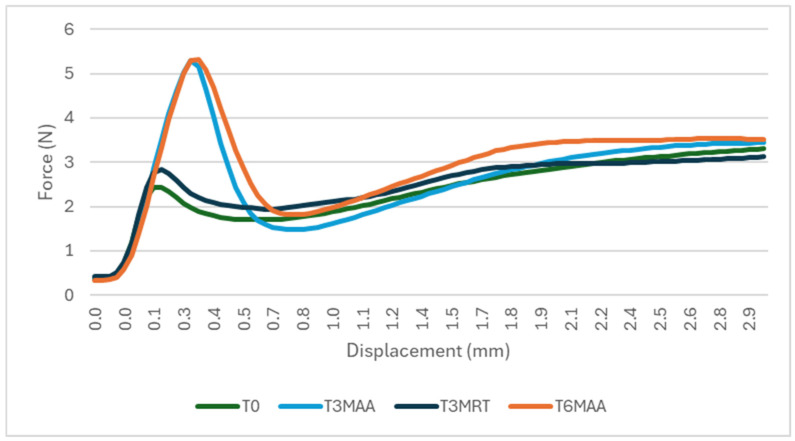
Break loose profile. Variable simulated drug-dependent test, 10 samples at each timepoint, specification: ≤10 N.

Break loose and extrusion forces were measured on the samples containing the surrogate solution. Figure 7 shows the entire measurement, while Figure 8 zooms into the break loose profile. One outlier measurement showed significantly higher force; it is excluded from the graph below, and the root cause has not yet been identified. The mean value of the 10 samples per each timepoint is shown.

### 3.6. Container Closure Integrity

Container closure integrity was measured in four variations (Table 8, Table 9, Table 10 and Table 11) to prove integrity of the closure system (closure system liquid leakage), integrity of the plunger (liquid leakage at the plunger), and the entire system (with helium leak as deterministic method and with blue dye test as probabilistic method [24]). Samples tested were 59 for the water solution and 15 and 12, respectively, for the surrogate solution. All tests passed (Table 8, Table 9, Table 10 and Table 11).

#### 3.6.1. Closure System Liquid Leakage

Closure system liquid leakage was measured with water and surrogate solution as an attribute test, using 59 and 15 samples, respectively. None of the syringes showed droplets (Table 8).

**Table 8:** Closure system liquid leakage.

Timepoints	Droplets/Total Number of Samples	
	Water	Surrogate Solution
T0	0/59	0/15
T3MRT	-	0/15
T3MAA	-	0/15
T6MRT	0/59	-
T6MAA	-	0/15
T1YAA	0/59	-

#### 3.6.2. Liquid Leakage at the Plunger

Liquid leakage at the plunger was measured with water and surrogate solution as an attribute test, using 59 and 15 samples, respectively. None of the syringes showed droplets (Table 9).

**Table 9:** Liquid leakage at the plunger.

Timepoints	Droplets/Total Number of Samples	
	Water	Surrogate Solution
T0	0/59	0/15
T3MRT	-	0/15
T3MAA	-	0/15
T6MRT	0/59	-
T6MAA	-	0/15
T1YAA	0/59	-

#### 3.6.3. Container Closure Integrity at the Plunger

Container closure integrity at the plunger was categorized as variable data resulting in a sample size of 45 samples. All syringes were below the Kirsch limit (Table 10).

**Table 10:** Container closure integrity at the plunger.

Timepoints	Failure Above Kirsch Limit/Total Number of Samples	
	Water	Surrogate Solution
T0	0/45	0/12
T3MRT	-	0/12
T3MAA	-	0/12
T6MRT	0/45	-
T6MAA	-	0/12
T1YAA	0/45	-

#### 3.6.4. Dye Ingress (Tightness Test)

Dye ingress was only measured with the surrogate solution as an attribute test, using 12 samples. None of the syringes showed a color change (Table 11).

**Table 11:** Dye ingress (tightness test).

Timepoints	Color Change/Total Number of Samples
	Surrogate Solution
T0	0/12
T3MRT	0/12
T3MAA	0/12
T6MRT	-
T6MAA	0/12
T1YAA	-

### 3.7. Deliverable Volume

**Figure 9:**
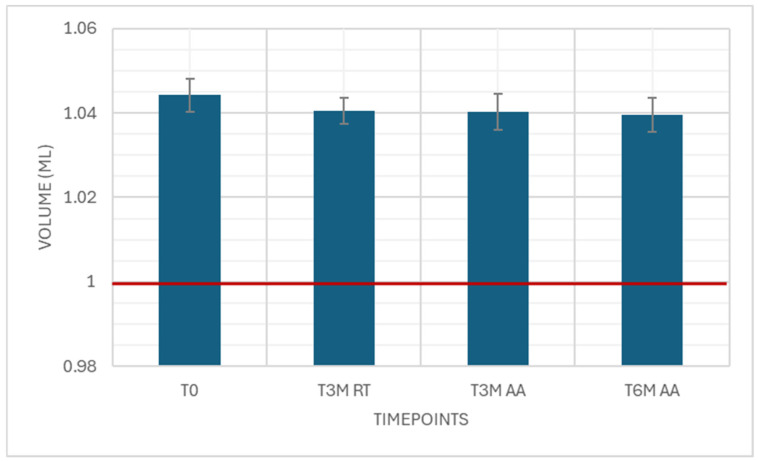
Deliverable volume. Attribute simulated drug-dependent test, 15 samples each timepoint, specification ≥ 1.00 mL.

The deliverable volume was measured as an attribute test with five samples using water. As the results are not expected to change, only T0 was measured. All samples passed. With the surrogate solutions, 15 samples and all timepoints were measured. The results were well above the limit of 1.00 mL (Figure 9).

### 3.8. Residual Volume

**Figure 10:**
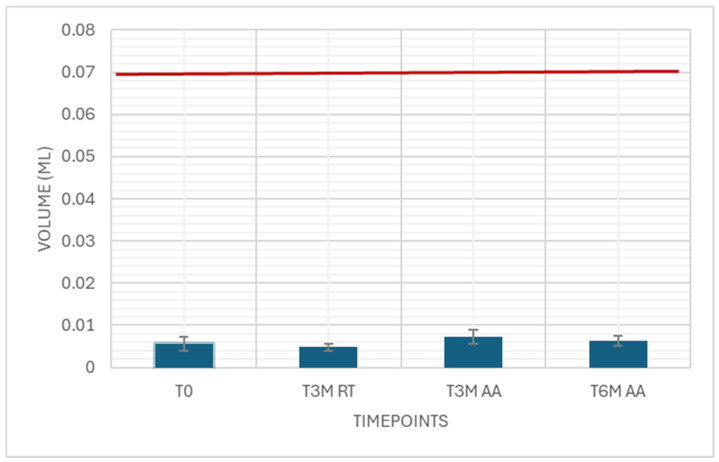
Residual volume. Attribute simulated drug-dependent test, 15 samples each timepoint, specification ≤ 0.07 mL.

The residual volume was measured as an attribute test with 59 samples using water. As the results are not expected to change, only T0 was measured. All samples passed. With the surrogate solutions, 15 samples and all timepoints were measured. The results were well below the limit of 0.07 mL (Figure 10).

## 4. Discussion

This case study contributes a practical DV template that links intended use → user requirements → QTPP → design inputs/outputs → EDDOs → DV tests and acceptance criteria, enabling transparent traceability beyond a “test list” approach. A pragmatic sample size rationale is provided for DV endpoints with mixed data types (variable vs. attribute), illustrating how confidence/reliability targets and distributional assumptions can be combined into a risk-commensurate sampling strategy.

Across the evaluated endpoints, results met the predefined acceptance criteria under the applied test conditions and timepoints. For a final evaluation, further aspects may be considered for each parameter, as described below.

Needle shield pull-off force in this case study was found to be towards the lower end of the specification. This must be interpreted in the context of rigid needle shield design (material, inner geometry, venting features), the rubber/needle lubrication state, and dimensional tolerances of the needle and shield interface, as well as the glass syringe tip/cone geometry. In addition, storage/aging conditions (temperature, time), sterilization effects, and the needle shield assembly method (push-on force, alignment) can materially shift removal forces. Both limits are critical here as needle shields should not be too loose to easily fall off, nor too tight so they cannot be removed without excessive force.

Needle pull-out force is strongly driven by the process of attaching the needle (type and amount of adhesive, thermal staking parameters) and the syringe cone/needle seat geometry and tolerances. It should also be assessed with consideration of mechanical stresses from handling, packaging and transportation. The consistent results show that the needle pull-out force is not impacted by aging.

Needle penetration force depends primarily on needle geometry (gauge, bevel type, sharpness, tip quality) and surface condition (silicone oil coating, burrs). These results are also highly consistent, showing independence from aging.

Flange breakage resistance must be considered against the syringe forming process, annealing quality, and dimensional features such as flange thickness, radius and surface defects that drive stress concentration. Handling history (nesting/denesting forces, transport shock), environmental conditioning, and test fixture alignment/loading rate significantly affect the surface quality of the glass and thus the breakage resistance. This is also shown in the higher variability of the measured results. Nonetheless, the results are far beyond the specification. As breakage resistance is highly dependent on handling, these results can only be valid for the measured samples, and it is essential to ensure an appropriate process is in place to keep the surface defects as low as possible. Very good insights into appropriate glass handling can be found in the PDA Technical Report No. 87 [25].

While the break loose force in this case study was consistently low in the empty samples, there was a rise seen in the 3 and 6 months accelerated aging samples, but not in the 3 months real-time aging samples. Both break loose and glide force depend on the siliconization strategy (type, amount plus tolerances, distribution, potential migration, application method), elastomer formulation/lamination/coating, syringe barrel inner diameter and tolerances, and plunger placement method (e.g., vent tube vs. vacuum placement, insertion speed, applied force, and compression set). Additional influences include barrel inner diameter tolerance, storage orientation, temperature, and aging timepoints, all of which can change static friction at the start of motion. Process factors such as plunger setting depth, hold time before testing, and shipping-induced micro movements can further alter the force displacement profile and its variability. Extrusion force integrates plunger–barrel friction with formulation-driven flow resistance, and therefore depends on drug viscosity (and its temperature dependence), needle gauge/length, needle internal roughness, and any constrictions in the flow path, as well as potential time-dependent effects (e.g., protein adsorption, silicone oil migration, drug-silicone oil interactions) that can change friction or flow over shelf life. Extrusion forces in all samples showed a high consistency over aging.

Tightness, liquid leakage (at the front closure and the plunger), and deterministic CCI results should be interpreted as a combined picture of sealing performance, because they probe overlapping interfaces (plunger–barrel seal and front-end/needle-shield region) under different driving forces and detection sensitivities. Key influencing factors include component tolerances and surface conditions, plunger placement depth/method and resulting compression set, temperature, and internal pressure/headspace effects. Handling, sterilization, transport and aging can further introduce micro defects or relaxation effects that shift results over shelf life. All results passed with no leakage.

Deliverable and residual volume should be evaluated together because they are coupled through fill volume tolerance and system dead space, which is driven by syringe cone/needle/shoulder geometry, plunger head design and deformation at end-of-stroke. In addition, actuation conditions (speed, hold time at end-of-stroke), temperature, and formulation properties (viscosity, wetting/film formation) can shift the balance between expelled volume and retained volume, and should be controlled and reported when interpreting results across lots and aging timepoints. Results shown are highly consistent and independent of aging, as expected.

## 5. Conclusions

This paper presents a risk-based design verification framework for a 1 mL long glass prefilled syringe system used as a device constituent part for subcutaneous delivery, linking intended use, user requirements and QTPP elements to measurable design outputs and a focused set of EDDOs. To demonstrate how this framework is applied in practice, the complete workflow was executed using a 1 mL long glass PFS filled with a surrogate solution, and the corresponding measurement approach and results across functional, mechanical and integrity endpoints were reported. In this way, the case study is intended not as an isolated dataset, but as an end-to-end example showing how the proposed template is operationalized: from requirement definition through test execution to evidence generation. Under the conditions and timepoints evaluated in this case study, all DV results met predefined acceptance criteria, supporting fit-for-purpose performance of the syringe system in terms of preparation and handling, dose delivery functionality, mechanical robustness, and container closure integrity.

The approach is transferable to other PFS programs, as well as other combination products, and can serve as a practical template to:

1. structure DV test selection;
2. define sampling plans commensurate with risk;
3. transparently justify acceptance criteria when standards and pharmacopeias primarily define finished-product expectations rather than component-level limits.

As a regulatory expectation, continued real-time aging to the end of shelf life and drug-dependent confirmation with the commercial formulation are the next steps to complete the evidence package.

Finally, we would also like to share some practical lessons learned. Early identification of QTPP elements is critical, as it drives downstream decisions and changing the QTPP late in development can necessitate re-definition of requirements and repetition of DV activities, hence the importance of “beginning with the end in mind.” Time and aging can affect endpoints differently; therefore, bracketing and timepoint selection should be tailored to the specific performance attribute rather than applied uniformly. Sampling plans should be defined as risk-commensurate decisions informed by formal risk evaluation and the data type (attribute vs. variable), rather than by a single universal convention. Finally, systematic generation and use of industry benchmarking datasets, through studies such as the present work, can support more transparent, evidence-based acceptance criteria settings where component-level limits are not harmonized across standards.

## Figures and Tables

**Table 1:** Exemplary extract of user needs and user requirements for a PFS administered by HCPs.

Requirement Attribute	User Needs	User Requirements
Usability	The user needs to exert minimal injection force for manual push	The device must enable manual injection with a maximum required force of ≤30 N
Functionality	The user needs to be able to accurately deliver the prescribed dose	The device must deliver the prescribed volume (e.g., 1.0 mL ± 0.1 mL) under intended use conditions
Reliability	The user needs to rely on drug delivery with a low device failure rate	The device must consistently deliver the full dose with a device failure rate below established reliability thresholds (e.g., ≥95% reliability at release)

**Table 2:** Exemplary extract of relevant elements from a Quality Target Product Profile (QTPP).

QTPP Attribute	QTPP Target
Dosage form, route of administration	Liquid, single use, subcutaneous injection
Container closure system	1 mL long staked needle prefilled syringe
Dose strength and volume	100 mg fixed dose, given in 1 mL
Viscosity	≤20 cP at 20 °C
Stability	Shelf life of ≥2 years at 2–8 °C
Safety	Impurities and leachables below safety threshold, no local/systemic adverse biological response

**Table 3:** Exemplary extract of design inputs and corresponding design outputs for the device constituent part.

Requirement Attribute	Design Input Criteria	Design Outputs
Functionality and Reliability	Device must not deliver less than 1.0 mL	Syringe barrel and plunger drawing, dimensional specification and tolerances; production process control strategy
Usability	Break loose force must not exceed 10 N	Silicone oil specification and analytical method; plunger/barrel interference fit
Usability	Extrusion force must not exceed 20 N	As above; needle gauge specification; formulation viscosity range
Drug stability	CCI must be given Contamination control	As above; contamination control strategy
Biocompatibility	Must comply with ISO 10993-1 [13]	Biocompatibility assessment

**Table 4:** Exemplary Essential Drug Delivery Outputs (EDDOs).

EDDO	Acceptance Criterion	Linked User Need/Risk
Deliverable volume	Not less than 0.9 mL under intended use conditions	Accurate dosing; risk of underdosing
Break loose force	Break loose force ≤ 10 N	Ability of user to complete injection; risk of incomplete injection
Extrusion force	Extrusion force ≤ 20 N	Ability of user to initiate injection; inability to deliver the dosage

**Table 5:** Exemplary extract of the device constituent part specification.

Test	Test Method Reference	Specification	Specification References
Needle shield pull-off force	ISO 11040-4 [14]	≥2 N and ≤35 N	Usability engineering and benchmarking
Needle pull-out force	ISO 11040-4	≥22 N	ISO 11040-4:2024
Needle penetration	ISO 11040-4	≤3 N	ISO 11040-4:2024
Flange breakage resistance	ISO 11040-4	≥35 N	ISO 11040-4:2024
Break loose force	ISO 11040-4	≤10 N	Usability engineering; intended user population + risk assessment; benchmarking
Glide force	ISO 11040-4	≤5 N	Engineering studies on friction performance (siliconization/plunger fit) + risk assessment; benchmarking
Extrusion force	ISO 11040-8	≤20 N	Intended user capability and formulation/needle flow resistance; benchmarking
Tightness test (blue dye)	ISO 11040-4	No color change	ISO 11040-4:2024
Closure system liquid leakage	ISO 11040-4	No droplets are visible around the external surfaces of the RNS	ISO 11040-4:2024
Container closure integrity	ISO 11040-8	≤6.0 × 10^−6^ mbar·L/s (Kirsch’s limit) [15]	MALL * strategy/microbial ingress correlation
Liquid leakage at the plunger	ISO 11040-8	No leakage found past the last seal of the plunger	ISO 11040-8:2025
Deliverable volume	USP <697>	Not less than 1 mL	USP <697>
Residual volume	ISO 7886-1 [16]	≤0.07 mL	ISO 7886-1

* Maximum allowable leakage limit.

**Table 6:** Overview of DV test matrix for both drug-independent and drug-dependent testing, including categorization and sample size; “(in)dependent” indicates that it is both drug-independent and drug-dependent.

Test	Drug-Dependent/ Drug-Independent	Attribute/Variable	Confidence/ Reliability Level %	Samples
Needle shield pull-off force	Independent	Variable	95/95	40
Needle pull-out force	Independent	Variable	95/95	40
Needle penetration	Independent	Variable	95/90	35
Flange breakage resistance	Independent *	Variable	95/95	40
Break loose force	(In)dependent	Variable	95/95	40
Glide force	Independent	Variable	95/95	40
Extrusion force	Dependent	Variable	95/95	40
Tightness test	(In)dependent	Attribute	95/95	59
Closure system liquid leakage	(In)dependent	Attribute	95/95	59
Container closure integrity	(In)dependent	Variable	95/99	45
Liquid leakage at the plunger	(In)dependent	Attribute	95/95	59
Deliverable volume	(In)dependent	Attribute	N/A	5 **
Residual volume	(In)dependent	Attribute	95/95	59

* Strongly process-dependent; ** as per USP <697> [20], N/A: Not applicable.

**Table 7:** Overview of DV test matrix for both drug-independent and simulated drug-dependent test, including sample size and timepoints.

Test	Drug-Independent Sample Size/Timepoints	Drug-Dependent Sample Size/Timepoints
Needle shield pull-off force	40/T0, T6MRT, T1YAA, T1YRT	-
Needle pull-out force	40/T0, T6MRT, T1YAA, T1YRT	-
Needle penetration	35/T0, T6MRT, T1YAA, T1YRT	-
Flange breakage resistance	40/T0, T6MRT, T1YAA, T1YRT	-
Break loose force	40/T0, T6MRT, T1YAA	10/T0, T3MAA, T3MRT, T6MAA
Glide force	40/T0, T6MRT, T1YAA	-
Extrusion force	-	10/T0, T3MAA, T3MRT, T6MAA
Tightness test	-	12/T0, T3MAA, T3MRT, T6MAA
Closure system liquid leakage	59/T0, T6MRT, T1YAA	15/T0, T3MAA, T3MRT, T6MAA
Container closure integrity	45/T0, T6MRT, T1YAA	12/T0, T3MAA, T3MRT, T6MAA
Liquid leakage at the plunger	59/T0, T6MRT, T1YAA	15/T0, T3MAA, T3MRT, T6MAA
Deliverable volume	5/T0	15/T0, T3MAA, T3MRT, T6MAA
Residual volume	59/T0	15/T0, T3MAA, T3MRT, T6MAA

## Data Availability

The original contributions presented in this study are included in the article. Further inquiries can be directed to the corresponding author.
